# Do book consumers discriminate against Black, female, or young authors?

**DOI:** 10.1371/journal.pone.0267537

**Published:** 2022-06-13

**Authors:** Dana B. Weinberg, Adam Kapelner

**Affiliations:** 1 Department of Sociology, Queens College, CUNY, Queens, NY, United States of America; 2 Department of Mathematics, Queens College, CUNY, Queens, NY, United States of America; TED University, TURKEY

## Abstract

The publishing industry shows marked evidence of both gender and racial discrimination. A rational explanation for this difference in treatment of both female and Black authors might relate to the taste-based preferences of book consumers, who might be less willing to pay for books by such authors. We ran a randomized experiment to test for the presence of discriminatory preferences by consumers based on authors’ race, gender and/or age. We collected ratings of 25,201 book surveys across 9,072 subjects on Amazon’s Mechanical Turk, making this study the largest experimental study of the book market to date. Subjects were presented with mocked-up book covers and descriptions from each of 14 fiction and non-fiction genres, with one of three possible titles per book randomly assigned. Using author names and photographs, we signaled authors’ race, gender, and age and randomly assigned these combinations to each book presented to our subjects. We then asked subjects to rate their interest in purchasing the book, their evaluation of the author’s credentials, and the amount they were willing to pay for the book. The experimental design of this study strived to eliminate the potential for proxy-based discrimination by providing book descriptions that detailed the authors’ relevant professional experience. The large sample allowed for exploration of various types of taste-based discrimination observed in the literature, including discrimination against particular groups, homophily, and pro-social behavior. Overall, book consumers showed a willingness to pay approximately $0.50 or 3.5% more on average for books by Black authors and little, if any, practically meaningful discrimination based on age or gender. In other words, our study finds no and even contrary evidence of taste-based preferences by consumers that would rationalize the historic discriminatory treatment of Black or of female authors by publishers nor of discrimination based on an author’s age.

## Introduction

The Black Lives Matter protests in the summer of 2020 called attention to systemic racism in American society. In the #PublishingPaidMe protest on Twitter, authors shared the amount of their advances and in so doing revealed the pay discrimination for Black authors, who tend to receive lower advances on their books than their White counterparts [1]. Close scrutiny of the industry highlighted its whiteness, not only in terms of authors and who receives recognition but also in terms of editors and decision-makers [1–3]. These trends have deep historical roots, but little if anything had previously been accomplished in addressing these trends in recent years [3]. However, following the BLM protests and #PublishingPaidMe, over a thousand people in the publishing industry signed up for a day of action to support Black authors, and publishers made statements of support for racial justice, announced antiracism workforce training, and pledged to publish more books by writers of color [1]. These conversations in publishing echo ongoing discussions about gender inequality in the industry, which similarly point to disparities in who gets published, who gets reviewed in prestigious outlets, and how much authors are paid for their work (see for example the annual VIDA counts and their publications [4]). Despite the attention these conversations got alongside #MeToo, particularly in 2018, it is unclear that these conversations have spurred any meaningful or lasting change.

An historic cultural gatekeeper, publishing has become increasingly profit-focused [5]. While editors purportedly used to call the shots based on taste and cultural importance, acquisition decisions and investments in particular book projects have increasingly become the purview of marketing departments [5]. Decisions about advances, advertising budgets, and other decisions about book production and distribution are based on expectations of a book’s or an author’s performance in the market [6]. Such organizational logics have historically been used to justify the lower pay and book prices for women compared to men [6]. However, publishers have also played an active role in creating and cultivating markets and crafting their expectations about book pricing, as in the structure of the female dominated romance market which focuses on mass market production of inexpensive books by women for women [7]. In attempts to diversify the racial and ethnic diversity of their offerings, publishers have tended to create specific and typically niche imprints for these works. Perhaps publishers have thus created their own self-fulfilling prophecies about anticipated performance and market behavior by marketing to specific and limited audiences and by making investment choices that both signal a lower investment in these works and give them less opportunity for discovery by a broader public.

However, publishers, for all of their shortcomings, are not the only potential source of discrimination in the book industry. With the closing of brick-and-mortar chains and independent bookstores as well as the shift in the product offerings within these venues, traditional publishing has become increasingly platform-based in its sales. Etailers like Amazon dominate the sales market both for digital and physical books. Unlike brick and mortar stores which have limited shelf space, online retailers can carry an almost unlimited number of titles. Whether those titles come from traditional publishers or from self-published authors, also known as “indie authors”, the etailers’ platform algorithms play a dominant role in product visibility. To the extent that these ranking and visibility algorithms incorporate consumer ratings and purchases, these algorithms may also be influenced by consumers’ discriminatory behavior and preferences. Yet consumer ratings are currently exempt from regulation and protection against discrimination [8, 9] and immune to publishers’ antiracist institutional practices. Moreover, to the extent that publishers use these ratings and algorithmic visibility in decision-making about which authors to publish in the future and how much to invest in their titles, such external evaluations provide a ready way to “launder” discrimination [10].

We further see the potential for discrimination from sources other than publishers when we consider the case of indie (self) publishing. Indie publishing has removed the gatekeeping and curation function played by publishers. An example of the gig economy or platform-based economy, indie publishing enables authors to market directly to consumers without the mediation of a publisher. On the one hand, this arrangement has the potential to remove the unconscious biases and prejudices of publishers that contribute to systemic racism or sexism in their acquisitions, production, distribution, and promotion of their catalogs. On the other hand, the consumer-facing gig economy offers no protections to authors from the potential discrimination by consumers and the potential ripple effect of that discrimination in the rating and visibility of their titles. Thus, the gig economy may prove more egalitarian given the removal of barriers to entering the market, but it may also heighten discrimination in ways that exacerbate inequality.

In short, in order to understand discrimination in the book industry, we must consider not only the behavior of publishers but also the behavior and preferences of consumers. This study uses a large-scale, randomized field experiment of over nine thousand subjects to examine the effects of author race, alongside gender and age, on consumers’ stated interest in a given book, their evaluation of an author’s credentials, and the prices consumers report they are willing to pay for books in a variety of fiction and non-fiction genres.

### Correspondence studies and patterns of discrimination in employment and product markets

Correspondence studies have been used to investigate discrimination, with a preponderance of studies examining discrimination in labor and housing markets [11, 12]. The classic study design sends resumes in response to job ads or real estate listings from equally qualified candidates who differ most commonly by race [11], although studies have also examined gender and age as well as the intersection of these attributes [12–14]. In a similar tradition, recent studies use experimental techniques to study discrimination in product markets: for example, Airbnb rentals [15], iPod sales [16], baseball card sales [17], and online car sales [18] (see [11, 19] for reviews). Correspondence studies have also been used to study differences in evaluation, particularly of writing, academic work, or creative products. Following the touchstone study by Goldberg [20], most evaluative correspondence studies have been designed to examine unconscious gender bias. Typically, names are used to signal race and gender, while graduation and employment dates are used to signal age. However, product market studies on digital platforms have also used photographs of subjects with different skin tones to signal race (for example, [16–18]).

Correspondence studies that find evidence of discrimination in product markets tend to find one of three patterns of inequality: 1) discrimination against particular groups, 2) homophily toward the consumers’ ingroup, 3) pro-social behavior toward the consumers’ outgroup.

#### Discrimination against particular groups

Reviews of correspondence studies related to race find stable evidence of discrimination against racial and ethnic minorities, particularly by employers [11, 21, 22]. These studies tend to focus on whether or not candidates are called for interviews and have found evidence of discrimination during the written stage involving resume vetting [11]. Very few studies follow through to collect data on job offers and differences in pay. Similar evidence of discrimination has also been found in experiments on product markets. Doleac and Stein [16] conducted an experiment in 2009 with hands of different skin tones holding iPods to denote the seller’s race; Black sellers received 13% fewer responses and 17% fewer offers than White sellers. Edelman et al.’s [15] Airbnb study used visitor profiles and looked at decisions on whether or not unit-holders rented to candidates based on race (determined by name), with some analysis of gender as well. They found evidence of racial discrimination by owners that had not previously rented to Black customers.

Correspondence studies related to gender show a more nuanced set of results as discrimination tends to depend not only on gender but on the gender stereotypes related to work or product markets. In a meta-analysis of correspondence studies related to labor market callbacks and gender, Birkelund et al. [13] find a consistent rate of callbacks for men across occupations, regardless of gender distribution. However, taking into account the gender distribution of particular occupations, they find that women receive more callbacks in female-dominated and integrated occupations but fewer in male-dominated occupations.

Experiments on gender-based differences in the evaluation of the work or qualifications of male and female workers also tend to show discrimination against women (for a review see [23]), especially in relation to the gender distribution in a given field or product market. Experiments using identical resumes have found disadvantages for female candidates in evaluations of their suitability for academic and scientific jobs [23–25], which are stereotypically male. Taking into consideration the stereotypes attached to particular product markets, Tak, Correll, and Soule [26] examined ratings of craft beer and cupcakes when producers were thought to be either male or female and found evidence of asymmetric negative bias: products made by women are disadvantaged in male-product markets (beer) while products by men suffer no disadvantage in female-product markets (cupcakes). However, other studies have found gender disparities irrespective of the product market perceptions. For example, Kricheli-Katz and Regev [27] find that buyers were willing to pay less for money-value gift cards sold by women than those sold by men.

Several correspondence studies have found discrimination against older workers, particularly older female job candidates [12], but the age categories are not comparable across studies. For example, while Albert et al. found discrimination against job candidates older than 38 [28], Richardson et al. [29] found employers considered candidates aged 42–48 more competent and more likely to be hired compared to both younger and older candidates, with the strongest discrimination against candidates over age 54. In a similar vein, at the intersection of age and race, Lahey and Oxley [14] found that job screeners discriminated against Black job candidates, but this discrimination was less against middle aged candidates than younger or older ones.

#### Homophily

Another pattern of discrimination found in the research literature is homophily, wherein experimental subjects show favoritism to members of their own ingroup. Nunley et al. [30] set up 288 auctions on eBay with matched pairs of sellers selling the identical products, using names to signal whether the seller was Black or White. They found higher prices paid to same-race seller (higher prices to sellers of buyers’ own race), in other words, evidence of homophily. However, they attributed the observed pattern of discrimination to lack of information about a seller’s credibility and found no statistically significant price differences when there was sufficient buyer feedback to establish seller credibility. Zigerell’s [19] meta-analysis of seventeen experimental studies that allow for investigation of racial discrimination uncovered a pattern of net discrimination in favor of Black targets by Black study participants, indicating a statistically significant pattern of homophily among Black participants when pooled across studies. Similarly, Edelman et al.’s [15] AirBnb study found homophily among Black female renters and rentees, though not among other groups. In an experimental study of manuscript reviews, Lloyd [31] found that female reviewers accepted articles by female authors significantly more frequently than did male reviewers (62% vs 21% acceptance of articles by female authors) and were more likely to accept manuscripts by female authors than male authors (62% vs 10%).

#### Pro-social behavior or social response bias

Finally, results from other studies suggest the possibility of pro-social behavior, wherein ingroups give more favorable ratings to outgroups than to ingroup members. For example, Asad et al. [32] found strong evidence of “race based altruism,” wherein white workers worked harder to generate income for black employers in an experiment that used images of hands with different skin tones to signal an employer’s race. In relation to age, while Weiss and Maurer [33] found no evidence of discrimination in favor of older workers in their replication of a 1970’s study, they found that their sample of student evaluators did not discriminate against older workers. One explanation proferred related to potential social response bias:

A final possible explanation is that students of today are less willing to express negative feelings toward older workers. In today’s diversity-conscious society, the expression of attitudes that are seen as negative toward a certain group is almost certainly unacceptable. The students used in our sample have been exposed to this kind of atmosphere for the larger portion of their lives and may have developed social desirability filters that keep them from expressing negative attitudes toward protected groups [33].

In short, there appears to be three distinct patterns of discrimination in the experimental literature. The first, discrimination against particular groups involves subject preferences that seem to be consistent across subjects, more or less independent of their own demographic characteristics. In contrast, other studies show patterns of discrimination that reflect interactions between the subjects’ demographic characteristics and the object of the experiment, reflecing either homophily (ingroup favoritism) or pro-social behavior (outgroup favoritism).

### Types of discrimination: Taste-based and proxy-based discrimination

Throughout the literature, especially coming from studies in Economics, there is a differentiation between taste-based discrimination and what we term “proxy-based” discrimination, encompassing both statistical discrimination and cognitive shortcuts based on stereotypes. Taste-based discrimination reflects animus or bias toward particular groups. In contrast, proxy-based discrimination treats race or gender or age—or some other characteristic, for example sexual orientation, weight, attractiveness, or disability—as a proxy for other, unobserved attributes of crucial importance to the decision at hand, for example worker productivity, the financial solvency or reliability of renters, or the credibility of sellers.

Statistical discrimination explanations seek to provide a rational actor justification for discriminatory decision-making, wherein decisions are not based on taste or preference for one group over another but instead use group membership as a proxy for the attributes of true interest. The idea behind statistical discrimination is that critical but unobserved attributes have different distributions among social groups—usually gender, age, or racial or ethnic groups [14, 34, 35] —– and decision-makers use this observable group identification to enhance their statistical odds of selecting candidates with the unobserved but desired attributes.

Another type of proxy-based discrimination relates to decision-making that relies on cognitive shortcuts. Under conditions of limited information, of low task attention, or of low value placed on the decision—for example, a one-off job or a low-paying one—decisionmakers may not bother pursuing or processing additional information even when it might be available, tending instead to take “cognitive shortcuts” or “heuristic cues” based on preexisting social categories, often closely related to stereotypes [36–38]. In theory, these cognitive shortcuts would differ from statistical discrimination in that they are based on social “common knowledge”, which may or may not be accurate, rather than on knowledge of underlying population distributions of certain attributes.

Arguably, once a study accounts for proxy-based discrimination, the remaining patterns of inequality in outcomes—to the extent any are observed—reflect taste-based discrimination. Researchers have attempted to address proxy-based discrimination by providing decisionmakers with easily and quickly accessible information relevant to their decisions. For example, in another study of Airbnb host behavior, Cui, Li, and Zhang [39] found that while (fictitious) guests with African-American sounding names were 19.2% less likely to be accepted than those with white-sounding names, guest profiles with positive peer-generated reviews showed no significant difference in acceptance rate for the two sets of guests. Similarly, Tjaden et al.’s [40] study of ridesharing in Germany found that discrimination based on name-derived ethnicity was reduced in the presence of positive driver ratings and reviews.

Proxy-based discrimination and taste-based discrimination ostensibly reflect different decision-making processes and motivation—enhanced statistical odds, less time and effort in decision-making, or unvarnished bias. However, the inequality in outcomes—i.e. who is hired, who is shown or rented an apartment, or how much is paid for a product—may nonetheless be the same without intervention, ultimately perpetuating social inequality in outcomes [11].

### Hypotheses about discrimination by book consumers

Our primary aim in this paper is to assess whether the underrepresentation of Black authors in publishing and their concentration in racially-focused niches reflect taste-based market preferences, making Black authors a poorer financial prospect for publishers unless they are catering to Black audiences. We also examine the role of author gender in this product market, and we also consider the role of author age. In previous research, we found gender discrimination in price setting by both traditional publishers and indie authors with work by authors with female names or in female-dominated genres priced lower than those with male names or in male-dominated genres [6]. In a follow-up experiment, we found no differences in prices consumers were willing to pay in relation to author gender, although our pilot study involved only two fiction genres [41]. In the current study, we examine a broad range of book genres. We also use visual signaling, in addition to the author name signals in our previous research, to explore not only responses to gender but also to race and age. Based on the conflicting patterns in our prior studies—of gender bias from publishing houses and its absence from consumers—we hypothesize that underrepresentation of Black authors reflects the biases of publishing houses with little basis in underlying market dynamics. We similarly anticipate an absence of gender and age discrimination in this product market.

In the case of books, book descriptions and information about the author are readily available for potential consumers to see. This information is available on a product page in the case of online sales and on book covers or jackets in the case of physical ones, often times alongside a photograph of the author. Moreover, in the case of traditionally published books compared to indie books, the publisher’s choice to publish the book also serves as a marker of the author’s achievement and recognition as well as of content curation. To the extent that discrimination is based on cognitive shortcuts or statistical discrimination, then the inclusion of these various signals about an author’s credentials and a book’s content would predict a mitigation of these types of proxy-based discrimination, such that any resulting discrimination would reflect taste-based discrimination or consumer preferences.

For taste-based discrimination based on race, gender, and/or age, we posit the null hypothesis:

*H*_0_: There does not exist taste-based discrimination in (a) consumers’ ratings of author credentials, (b) interest in purchasing a book, nor in (c) the price consumers are willing to pay for a book.

Against this null hypothesis, we test alternative hypotheses related to three distinct patterns of discrimination, namely whether there are (*H*_1_) overall patterns of discrimination against particular groups, (*H*_2_) of homophily (in-group favoritism), or (*H*_3_) of pro-social behavior (out-group favoritism) in relation to consumers’ ratings of author credentials, interests in purchasing books, or in the prices consumers are willing to pay.

## Materials and methods

### Experimental recruitment

This study examines consumer book-buying preferences related to authors’ age, gender, and race for various types of books. A total of 9,067 respondents were recruited to our randomized experiment via the use of Human Interaction Tasks (HITs) on Amazon’s Mechanical Turk (MTurk) platform. Participants were adults living in the United States. Respondents were surveyed about their perceptions related to the book cover and description of a book in one of fourteen fiction or non-fiction genres. In addition to book genre, our randomized factorial design varied author age, gender, and race for each book presented. Respondents were asked questions about their preferences for up to three books in different genres, all with different authors.

This study uses a natural field experiment design on MTurk. In contrast to “framed” or “laboratory” experiments where the subject knows they are part of a study and even volunteer for the opportunity, participants in natural field experiments do not know they are part of an experiment. This design feature of natural field experiments addresses both concerns about selection bias of participants and of bias in their behavior or responses [42]. By combining randomization and realism, the treatment effects measured in natural field experiments are both causal and broadly generalizable [42].

Our natural field experiment recruited participants from MTurk. MTurk is the largest easily-accessible online, task-based labor market, which employs between 75,000–150,000 Americans of which approximately 1,800 are available at any time [43]. We created our experimental task to be inconspicuous and to appear much like any other one-off MTurk market research survey task. This study’s HIT title was “Answer Some Questions for a Book Publisher”; the description was “We are eliciting your opinions about books for a market research study. We will ask you to examine book covers and book descriptions and then to offer your opinions about the book.”; and searchable keywords were “survey, questionnaire, poll, opinion and market research”. Surveys with these types of titles, descriptions, and keywords are among the most commonly performed HITs. Our natural field experiment thus uses the “experimenter-as-employer” design of Gneezy and List [44] within MTurk as in Chandler and Kapelner [45]. As such, this study did not inform participants that they were part of an experiment. Instead, we presented our MTurk requester as a real book publisher, and we presented our fictitious books as real books for market evaluation. The study received IRB exempt approval with waiver of documentation of informed consent from the Queens College-CUNY IRB.

We collected our data in the two months between July 2 and August 31, 2020 at $0.18 per HIT. Five subjects were dropped due to database corruption for a total number of first surveys 9,067. Each subject then completed two more surveys for a total number of 25,201 book ratings.

### Experimental design

We follow the model of the correspondence studies and product market experiments wherein different subject are presented with equivalent products that differ only by the candidate or seller’s key attributes, in this case the author’s age, gender, and race. As in studies that consider the gender distribution of occupations or product markets, we presented books from a range of popular genres selected to represent male-dominated genres, female-dominated genres, and mixed genres. We used authors’ names as signals of gender and age and photographs as signals of age, gender, and race as described below.

#### Book genre, covers, titles, and blurbs

**Fig 1:**
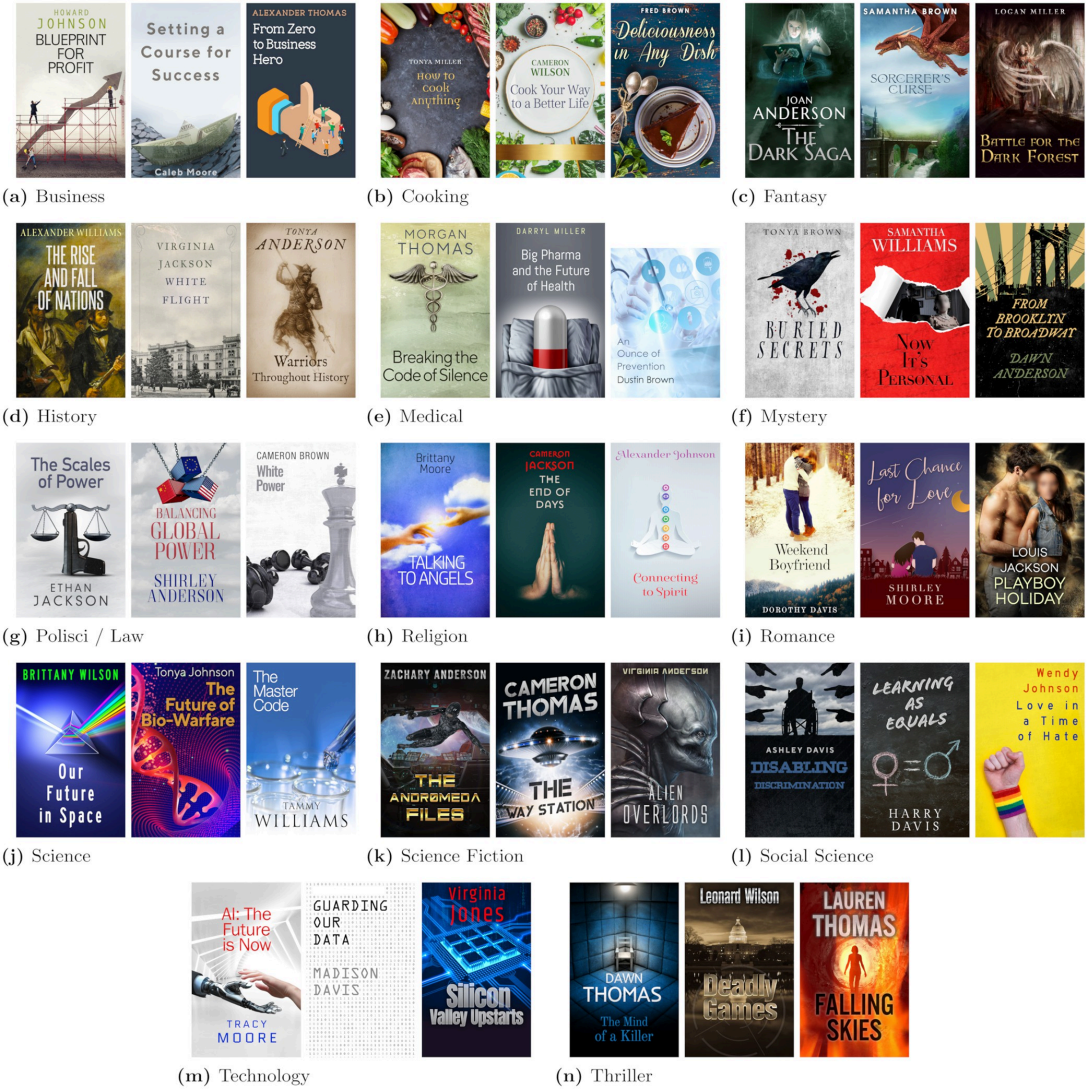
Cover art. The 42 cover art images used for the book cover manipulation with sample title and author combination selected at random to be displayed here. All covers are printed under a CC BY license with permission from Book Cover Zone, original copyright 2019. Faces in photos are blurred if they are potentially identifying.

We generated book covers and descriptions, or blurbs, for books in fourteen popular genres: Business, Cooking, Social Science, Law/Political Science, History, Religion, Medicine, Science, Technology, Science Fiction, Fantasy, Romance, Mystery, and Thrillers. We contracted with a professional cover design service, Book Cover Zone, to create three book covers representative of each genre for a total of 42 distinct covers. These covers are displayed in Fig 1.

**Fig 2:**
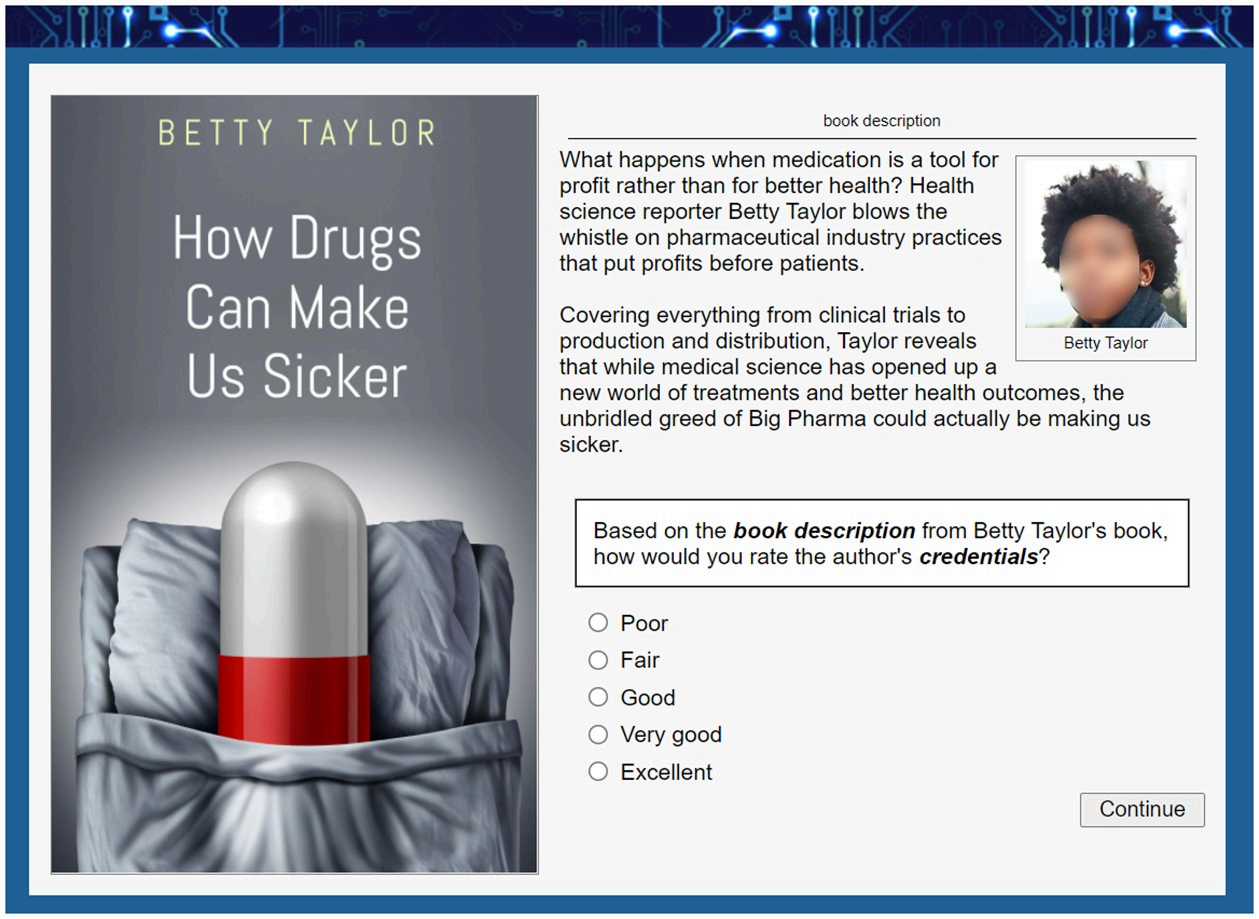
Survey page. An example “Author Credentials” survey response elicitation screen for the manipulation Black / Female / Millenial / Medical / Betty / Taylor. The blurb is found under “book description” and the survey question is located in the white box with answer choices below. The author headshot displayed here is a royalty-free image that was not employed in the experiment and blurred as the person would be identifiable. Our experiment had similar headshot photographs that cannot be released under a CC BY license. The cover image is printed under a CC BY license with permission from Book Cover Zone, original copyright 2019.

We mocked up book blurbs typical of titles in all of the genres that were reflective of the elements in the associated book cover art and reflective of the genre (by reading blurbs of current published offerings in each genre). The blurb always included the author’s full name (and if repeated, their first name) and a sentence or two about the author’s credentials, which were included to control for proxy-based discrimination. An example blurb can be found in Fig 2 and each of the 42 blurbs can be found in S2 Table.

We assigned one book blurb and three randomized titles for each cover (a total of 126 different book titles). Example titles can be found on the book cover renderings in Figs 1 and 2 and all 126 can be found in S1 Table.

#### Author names

We selected popular male and female author names to signal author gender. Kasof [46] observed that names also carry connotations regarding age, which could bias gender-related findings. We leverage this aspect of names to examine age-related preferences by selecting the one hundred most popular male and female names from the US Census for 1940 and 1950, 1960 and 1970, and 1980 and 1990, representing Baby Boomers, Gen Xers, and Millennials, respectively. We eliminated any names that crossed multiple generations as the goal was to select names that were characteristic of birth cohorts rather than classic names with sustained popularity. We then selected the remaining top nine names for males and females in each age category. The names for each gender and generation is shown in Table 1.

**Table 1:** Author first names. The nine manipulated author first names within each sex and age setting.

Generation	Sex	Names
Millennial	Male	Dylan Zachary Dustin Tyler Alexander Cameron Ethan Caleb Logan
	Female	Ashley Taylor Lauren Brittany Alexis Samantha Brianna Madison Morgan
Gen X	Male	Jeff Troy Chris Jon Dean Tim Darryl Greg Jamie
	Female	Tammy Tina Dawn Tracy Wendy Michele Jill Beth Tonya
Boomer	Male	Ralph Albert Eugene Fred Harry Howard Louis Stanley Leonard
	Female	Betty Shirley Dorothy Joan Gloria Virginia Marilyn Helen Ruth

For author last names, we used the fourteen most popular surnames from the 1990 Census [47]: Smith, Johnson, Williams, Jones, Brown, Davis, Miller, Wilson, Moore, Taylor, Anderson, Thomas, Jackson, White. We selected this year since the 1990 Census was likely to have included a cross-section from each of the respective generations under study. We randomly combined one name from each of our gender and age name categories with one of the last names from our list for each cover and title combination to render covers complete with author names (2 genders x 3 age categories x 14 genres x 3 covers and descriptions per genre X 3 titles per cover) for a total of 756 different cover, title, and author name combinations. Examples of all 42 covers rendered with one title and one author name can be found in Fig 1.

#### Author photographs

We had initially hoped to use names encoded for race, following the example of Bertrand and Mullainathan [48], “Are Emily and Greg More Employable Than Lakisha and Jamal: A Field Experiment on Labor Market Discrimination”. However, the most popular names overlap for American Whites and Blacks, and more racially-specific names were less socially prominent overall. Given that these more specific names might also signal other attributes that could bias findings, we instead opted to rely on photographs to convey an author’s racial phenotype together with age and gender.

We selected photo stock headshots and classified them according to White or Black phenotype, male or female gender, and age range of 20–39 (Millennial), 40–59 (GenX), or 60–79 (Boomer). One member of the research team did the initial coding. Two other coders were then asked to classify the same photographs. Only photographs with 100% inter-rater agreement on gender, age, and racial phenotype were included in this study. Enforcing uniformity on dimension and resolution as best as possible, we selected six photographs for each age, race, gender combination for a total of 6 × 3 × 2 × 2 = 72 different headshots to represent author avatars. The avatars were randomized in their presentation to respondents but matched with names corresponding to their age and gender classification.

Photographs were considered alongside covers and descriptions, similar to the way author avatars are presented beside books on Amazon and some other online e-tailers.

#### Subject allocation to author name, cover, title, and photograph

For each genre, cover and generation, we chose 3 first names and 3 last names (we explain this careful selection in the next paragraph) and thus rendered 3 unique name-stamped covers for a total of 14 genres × 3 covers per genre × 3 first names × 3 last names = 378 unique rendered images. For each of these images, we allocated 6 subjects per gender-race for a total of 2 genders × 2 races × 6 subjects = 24 subjects per image. Thus, the 9,072 subjects = 378 images × 24 subjects per image. The reason we have 6 subjects per gender-race cell is because they were each shown one of the six unique headshot photographs (see previous section). Thus each headshot is uniformly represented in the final dataset.

We now explain the randomization of the names within a genre. Within a gender-race-generation cell, each cover is represented three times for a total of nine unique images. The nine first names were randomly shuffled over these images. This means every first name is uniformly represented in the final dataset. For these nine images, nine unique last names were selected. The remaining five were left over for the next gender-race-generation cell. Because we randomized last name by block and 14 divides 9,072 evenly, each last name was also uniformly represented in the final data set.

The explicit manipulations for one genre tabulating the above logic can be found in S3 Table.

We were very careful to enforce uniformity over all manipulations in the first survey to ensure maximum power if we were to drop the two additional repeated measure surveys for each subject. For these two additional repeated measure surveys per subject, we did not enforce uniformity and opted to choose manipulations randomly with an important restriction: treatment genre, first name, last name and headshot photograph were unique over the three surveys any participant viewed.

### Consumer preferences

Most respondents were asked to rate three books (although those chosen for the post-manipulation check described below only rated one.) We used the following restrictions in the otherwise random assignment to avoid duplication: (1) the genre must be different thereby guaranteeing a different cover, (2) the author first name and last name must be different, (3) the author photo must be different than the others presented to the subject.

For each book respondents rated, they were asked about (1) their interest in the book based on the book description, (2) their evaluation of the author’s credentials, and (3) how much they were willing to pay for the book. We display the question text and the response variable values in Table 2. The elicitations appeared in a different random order for each survey for the purpose of minimizing order bias. Fig 2 shows a sample survey elicitation.

**Table 2:** Response variables. The three dependent variables measured in our study along with the specific question text and their possible measurement values.

Response Variable Name	Question Elicitation Text	Possible Responses (in $USD if numeric)
Author Credentials	Based on the ***book description*** from [Author’s full name]’s book, how would you rate the author’s ***credentials***?	Poor, Fair, Good, Very Good, Excellent
Purchase Interest	How interested would you be in ***buying*** this book?	Not at all interested, A little interested, Somewhat interested, Very interested
Willingness to Pay	What is the highest price ***you*** would be willing to pay for this book?	0, 0.99, 1.99, …, 48.99

### Respondent characteristics

We queried respondents’ readership preferences and demographic characteristics using a series of 14 questions. Each question appeared on a single screen (where moving back was not allowed) and appeared in a different random order for each subject in order to minimize order bias, a type of survey satisficing [49].

We asked respondents to report on various demographic characteristics, including: age, sex, race, ethnicity, urban environment, education, employment status, income, and marital status (see Table 3). We also asked respondents to report their state residency.

**Table 3:** Demographic characteristics of our experimental subjects.

Characteristic	Category	Percent	Average ± Std. Dev.
Age			35.6 ± 12.1
Sex	Female	55.2	
Race	White	73.1	
	Black	10.1	
	Asian	7.4	
	Amer. Indian	1.6	
	Pac. Isl.	0.2	
	Other	3.2	
	Mixed	4.5	
Hispanic Origin Environment	Yes	19.6	
	Urban	38.6	
	Suburban	45.0	
	Rural	16.4	
Education	High School Graduate	10.3	
	Attended College	21.1	
	College Graduate	52.3	
	Advanced Degree	16.3	
Employment	Full time	55.7	
	Part Time	15.6	
	In School	8.1	
	Temporarily unemployed	5.4	
	Homemaker	5.2	
	Unemployed	5.1	
	Retired	2.7	
	Other	2.1	
Income (in $1000/yr)	≥ 200	2.0	
	150–199	3.8	
	100–149	10.2	
	80–99	10.1	
	60–79	15.3	
	40–59	23.1	
	20–39	20.9	
	≤ 19	10.4	
	No response	2.9	
Marital Status	Married	50.5	
	Single never married	32.8	
	Living with partner	9.6	
	Divorced	4.1	
	Separated	1.2	
	Widowed	0.1	
	No response	0.1	

We also asked respondents about their reading preferences. We asked them to report on the number of print books they had read in the past six months, the number of books they had bought in the past six months, book format preferences, and book genres they had read in the past six months (see Table 4).

**Table 4:** Readership characteristics of our experimental subjects. Note that the book genre assignment adds up to >100% as respondents were able to indicate multiple genres.

Characteristic	Category	Percent	Average ± Std. Dev.
# Print Books Read in past Six Months			9.3 ± 12.6
# Print Books Bought in past Six Months			5.9 ± 8.0
Book Format Preference	Paperback	34.6	
	Hardcover	31.8	
	E-book	26.0	
	Audiobook	6.7	
	Other	1.0	
Book Genres Read in past Six Months	Cooking	27.9	
	Mystery	30.5	
	History	38.2	
	Medical	17.2	
	Thriller	24.9	
	Technology	22.5	
	Science Fiction	26.9	
	Science (nonfiction)	26.1	
	Romance	24.5	
	Fantasy	30.0	
	Business	30.9	
	Religion	18.0	
	Social Science	20.6	
	Political Science / Law	14.1	

### Analysis

#### Generalizability to American book consumers

We use a battery of demographic and readership measures to assess the extent to which our experiment might generalize beyond our respondents to draw inference about American book consumers. We examine our demographic variables to explore the representativeness of our sample with regard to the US population and similar MTurk studies. We also examine readership habits to establish the extent to which our respondents are both readers and book buyers.

#### Post-manipulation checks

We explicitly refrain from asking questions about these author characteristics in the survey as our aim is to capture unconscious bias. Consequently, the internal validity of our results relies on our subjects’ conscientious internalization of our experimental manipulations i.e. the subjects have to notice and mentally register the race, gender, age of the authors and the genre of the book in order to determine a response. One way of ascertaining the degree of this internalization is to query the race, gender, and age of the author and the book genre after the manipulation.

Such “post manipulation checks” (PMCs) have been recommended since the 1950’s [50, page 145], are common in social science experimentation [51, Table 1] and sometimes are required for grant-funded experimentation [52]. In order to not bias our subjects’ responses, nor reveal the experimental nature of our survey and nor reveal our research hypothesis [53], we placed our PMC at the end of the study after all responses were elicited. Since the PMC was measured directly after the response variables were captured, we do not believe its validity would be compromised by the subject forgetting the manipulation, a concern raised by Hauser et al. [51]. Furthermore, given the sequential nature of our study and the concern that MTurk subjects communicate with each other (i.e. a subject after the PMC who learns that our HIT is an experiment may intuit and subsequently expose our research hypothesis to other potential subjects), we chose to implement the PMC only for the last 1,000 HITs which were completed. This strategy limited the potential compromise of our experimental integrity. We have no reason to suspect there was any compromise as (1) the last 1,000 HITS were completed in the last seven days of our experiment and (2) we did not find any messages posted about our HIT on the popular MTurk public forums.

We analyze the PMC results using confusion matrices that compare the treatment received to respondents’ recalled perceptions about the book genre and author’s race, age, and gender presented to them.

#### Assessing patterns of discrimination

We fit four linear models to detect and describe patterns of discrimination in relation to our three response variables: ratings of book interest, ratings of an author’s credentials, and the amount the subject would be willing to pay for the book. We test for the main effects of our treatment with dummy variables for whether the book’s author was presented as Black (compared to White), female (compared to male), and GenX or Boomer (compared to Millennial). Model 1 shows only the main effects. Model 2 adds in covariates for book genre (with Business as the comparison) as well as the demographic and reader preference variables. With regard to demographic variables, the models shown in this paper show a parsimonious model, including only respondents’ age, gender, and race as none of the other demographic variables contributed substantively to model fit.

These two models investigate the presence of any discriminatory taste-based preference based on author age, race, or gender. To the extent that there are any patterns of discrimination against particular groups, the book-pricing and publication decisions of publishers would suggest discrimination against Black authors and female authors. In addition, given the knowledge-intensive nature of book writing, we might also anticipate age-based discrimination against Millennial authors.

Models 3 and 4 explore the interaction between author characteristics and respondents’ characteristics to test for both homophily (preference for ingroup, signified by a positive coefficient) and pro-social behavior (preference for outgroup, signified by a negative coefficient). Model 3 shows authors’ race, gender, and age; respondents’ race, gender, and age; and the respective interactions of author-respondent race, gender, and age. Model 4 additionally includes the covariates for genre and for readership preferences.

Our dataset of 25,201 book ratings is “clustered” [54, Chapter 2.1.1.1] on the individual MTurk respondents. Thus, we augment each model with random intercepts [54, Chapter 2.2] coded as the MTurk worker IDs resulting in a hierarchical mixed model. We then estimate the fixed effects of our experimental treatment, respondent demographics, and interactions via restricted maximum likelihood [54, Chapter 2.4.2].

## Results

### Sample description

Across our 9,072 unique American subjects, the sample breakdown was even across genres (648 surveys per genre); across covers (216 per cover); across race within cover (108 per cover-race cell); across gender within cover and race (54 per cover-race-gender); and across age within cover, race and gender (18 per cover-race-gender-age cell). This breakdown maximizes power on any of these manipulation variables interacted arbitrarily (assuming a homoskedastic linear model for the response metric under consideration).

#### Generalizability to American book consumers

To what extent might our experimental findings be generalizable to the population of American book consumers? An advantage of MTurk experiments over laboratory experiments is their greater potential for generalizability. The demographic results for our sample appear in Table 3. Longitudinal studies of MTurk respondents show stability in their self-reported demographic characteristics [55]. Our sample comports with previous studies of the MTurk population [56, Table 3 for example]. In addition, we examined the state within the United States that the subject reported residing (not shown) and found a correlation of 0.977 with the state-by-state population distribution according to the 2010 US Census data.

Another key factor in the external validity of this project is the extent to which our subjects are book consumers and, specifically, consumers of the genres presented in our study (i.e. there would be a heterogeneous treatment effect on the covariate “book consumption”). There is not reason for concern here. Table 4 shows that our subjects read approximately 1.5 print books monthly, representing a lower bound on their reading habits as the question excludes other formats of consumption like ebooks and audiobooks. Moreover, they buy approximately 1 book monthly and are avid readers in the 14 genres employed in this study.

These results combined with results from other MTurk experimental findings [56] provide reasonable evidence that the results herein can be generalized to American book consumers.

#### Post-manipulation checks

The MTurk respondents demonstrated sensitivity to our experimental treatments in our post-manipulation checks. Table 5 presents the results for the race, gender, and age manipulations as confusion matrices. The accuracy for the race manipulation PMC was 90.6%, for the gender manipulation PMC was 94.8%, and for the age manipulation PMC was 57.7%.

**Table 5:** Results of the post manipulation check (PMC) on the final 1,000 subjects in our experiment as confusion matrices. Rows indicate the true treatment manipulations and columns indicate the subjects’ responses. Accuracy tabulated in the last columns is the recall rate (in percent) and accuracy tabulated in the last row is the precision rate (in percent). DR indicates that the subject responded “I don’t remember”.

Actual ↓, Subject →	White	Black	DR	Other	Accuracy	Actual ↓, Subject →	Male	Female	DR	Accuracy
White	484	11	3	2	96.8	Male	479	22	3	95.0
Black	50	422	13	15	84.4	Female	25	469	2	94.6
Accuracy	90.6	97.5				Accuracy	95.0	95.5		
(a) Race PMC Results						(b) Sex PMC Results				
Actual ↓, Subject →	Millennial	GenX	Boomer	DR	Accuracy					
Millennial	246	73	1	20	72.4					
GenX	109	172	23	17	53.6					
Boomer	20	150	159	10	46.9					
Accuracy	65.6	43.5	86.9							
(c) Age PMC Results										

We can reasonably be assured that our manipulation for race and gender was effectively induced in the subject. However, for the age manipulation there were large errors in the proximal categories (Table 5c), but there was almost no error distinguishing Millennial from Boomer (the accuracy is 95.1%). Thus, we can at least trust our manipulation induced the desired effect on the subject when comparing these two age categories.

Although not critical to the objective of our study, we also provided a PMC for the genre manipulation. The accuracy here was 70.1% which is high given they were required to select one of 14 categories. The confusion matrix (unshown) shows errors that are expected among categories that traditionally have overlap in their offerings: technology is confused with business, science is confused with medical, political science is confused with social science, thriller is confused with mystery, and mystery is confused with thriller. This provides evidence that our cover art, blurb, and title induced the desired effect.

### Patterns of discrimination

The results of all four models for each of our response variables—author credentials, purchase interest and willingness to pay—can be found in Tables 6–8 respectively. Respondents rated their interest in buying the books at 1.35 ± 1.03, registering between “a little interested” and “somewhat interested” on a 4-point scale where zero represents no interest. They rated author credentials at 2.37 ± 1.00, representing between “good” and “very good”. Finally, they reported they were willing to pay $14.19 ± $10.86 on average.

**Table 6:** Rating of author qualification in the surveyed book.

	Coefficients and statistical significances			
Variable	Model I	Model II	Model III	Model IV
author race is black	0.11***	0.11***	0.11***	0.11***
author gender is female	0.02	0.02*	0.02	0.02
author generation is boomer	0.05***	0.05***	0.06***	0.06***
author generation is genX	0.04**	0.03**	0.04*	0.04**
author and subject race is black			-0.01	-0.01
author and subject gender is female			0.01	0.01
author and subject generation is boomer			-0.04	-0.05
author and subject generation is genX			-0.05	-0.05
book genre is cooking		0.23***		0.23***
book genre is fantasy		0.13***		0.13***
book genre is history		0.33***		0.33***
book genre is medical		0.23***		0.23***
book genre is mystery		0.23***		0.23***
book genre is polisci / law		0.13***		0.13***
book genre is religion		-0.06		-0.06
book genre is romance		0.05		0.05
book genre is science		0.32***		0.32***
book genre is science fiction		0.17***		0.17***
book genre is social science		0.10***		0.10***
book genre is technology		0.16***		0.16***
book genre is thriller		0.24***		0.24***
subject race is black		0.20***	0.21***	0.20***
subject gender is female		0.08***	0.08***	0.08***
subject generation is boomer		-0.04	-0.01	-0.01
subject generation is genX		-0.02	0.00	0.00
subject number print books read		0.00**		-0.00**
subject number print books bought		0.01***		0.01***
subject read genre past six months		0.10***		0.10***
(intercept)	2.27***	2.00***	2.21***	2.00***

Experimental coefficients are found above the horizontal line divider.

*** indicates *p* < 0.001,

** indicates *p* < 0.01 and

* indicates *p* < 0.05.

**Table 7:** Purchase interest in the surveyed book.

	Coefficients and statistical significances			
Variable	Model I	Model II	Model III	Model IV
author race is black	0.07***	0.07***	0.05***	0.06***
author gender is female	0.01	0.01	-0.01	-0.01
author generation is boomer	0.00	0.00	0.01	0.01
author generation is genX	0.01	0.01	0.01	0.01
author and subject race is black			0.10**	0.09*
author and subject gender is female			0.04	0.04
author and subject generation is boomer			-0.05	-0.04
author and subject generation is genX			-0.01	-0.02
book genre is cooking		0.30***		0.30***
book genre is fantasy		0.11***		0.11***
book genre is history		0.12***		0.12***
book genre is medical		0.21***		0.21***
book genre is mystery		0.36***		0.36***
book genre is polisci / law		0.15***		0.15***
book genre is religion		0.01		0.01
book genre is romance		-0.02		-0.02
book genre is science		0.17***		0.17***
book genre is science fiction		0.16***		0.16***
book genre is social science		0.18***		0.18***
book genre is technology		0.19***		0.19***
book genre is thriller		0.37***		0.37***
subject race is black		0.35***	0.32***	0.30***
subject gender is female		-0.09***	-0.12***	-0.11***
subject generation is boomer		-0.09**	-0.10*	-0.07
subject generation is genX		-0.01	-0.01	-0.00
subject number print books read		-0.01***		-0.01***
subject number print books bought		0.02***		0.02***
subject read genre past six months		0.38***		0.38***
(intercept)	1.30***	1.04***	1.34***	1.06***

Experimental coefficients are found above the horizontal line divider.

*** indicates *p* < 0.001,

** indicates *p* < 0.01 and

* indicates *p* < 0.05.

**Table 8:** Respondents’ willingness to pay for the surveyed book.

	Coefficients and statistical significances			
Variable	Model I	Model II	Model III	Model IV
author race is black	0.51***	0.50***	0.47***	0.47***
author gender is female	-0.11	-0.10	-0.19	-0.19
author generation is boomer	0.10	0.10	0.16	0.16
author generation is genX	0.08	0.08	0.11	0.12
author and subject race is black			0.31	0.28
author and subject gender is female			0.15	0.17
author and subject generation is boomer			-0.27	-0.25
author and subject generation is genX			-0.26	-0.22
book genre is cooking		1.35***		1.34***
book genre is fantasy		-0.80***		-0.80***
book genre is history		0.56**		0.57**
book genre is medical		1.29***		1.29***
book genre is mystery		0.48*		0.48*
book genre is polisci / law		0.66**		0.65**
book genre is religion		-1.37***		-1.38***
book genre is romance		-1.98***		-1.98***
book genre is science		1.15***		1.16***
book genre is science fiction		-0.56**		-0.56**
book genre is social science		-0.10		-0.10
book genre is technology		0.55*		0.54*
book genre is thriller		0.60**		0.60**
subject race is black		3.75***	3.79***	3.60***
subject gender is female		-1.17***	-1.41***	-1.25***
subject generation is boomer		-1.60***	-1.78***	-1.48***
subject generation is genX		-0.52*	-0.43	-0.40
subject number print books read		-0.11***		-0.11***
subject number print books bought		0.16***		0.16***
subject read genre past six months		1.46***		1.46***
(intercept)	13.88***	13.91***	14.46***	13.94***

Experimental coefficients are found above the horizontal line divider.

*** indicates *p* < 0.001,

** indicates *p* < 0.01 and

* indicates *p* < 0.05.

The main effects of the experiment are robust and stable across all four estimation models and are relatively unaffected by inclusion of additional covariates. Across all three response variables, consistent with our hypothesis, we do not find evidence of discrimination against Black or female authors. Rather, we find a robust result of significant taste-based preference in favor of Black authors. Though significant (all coefficients have p < 0.001), the differences related to evaluation are clinically small: less than a tenth of a point on the 4-point purchase interest measure and 0.11 of a point across all models on the 5-point author credentials measure. In contrast, respondents showed a substantial difference in willingness to pay of between $0.47 and $0.51 more for books by Black authors. This difference in willingness to pay translates to about 3.5% of the mean book price of $14.19.

Contrary to our hypothesis of no discrimination based on age, we do find significant (p < 0.001) favorable differences in the evaluations of Boomer and GenX authors’ credentials compared to Millennials. Again, however, these differences are clinically insubstantial, between 0.04 and 0.06 points on a 5-point scale. Moreover, they do not translate into differences in interest in purchasing the books or in what respondents are willing to pay.

Examining Models 3 and 4, which test the interaction between author and respondent characteristics, we only find significant effects for the interaction between Black authors and Black respondents with regard to purchase interest. We find slight evidence of homophily, wherein Black respondents express more purchase interest for Black authors’ books (0.09 points higher on a 4-point scale, p < 0.001). However, this result does not extend to evaluations of authors’ credentials or the amount respondents report they would be willing to pay for the books.

Given the lack of any other interaction effects, we find no further evidence of homophily nor of pro-social behavior.

## Discussion

The publishing industry shows marked evidence of both gender and racial discrimination. Female authors’ books have been consistently priced lower than those of their male counterparts, and female-dominated genres like romance have also been priced lower, regardless of author gender. In terms of race, book publishing has been characterized as a largely white industry, both in terms of the workforce in publishing houses and in terms of the race of published authors, and anecdotal evidence suggests Black authors receive lower advances for their work. A rational explanation for this difference in treatment of both female and Black authors might relate to the taste-based preferences of book consumers, who might be less willing to pay for books by such authors.

We ran a randomized experiment to test for the presence of discriminatory preferences by consumers based on authors’ race, gender and/or age. We collected ratings of 25,201 book surveys across 9,072 subjects on Amazon’s Mechanical Turk, making this study the largest experimental study of the book market to date. Subjects were presented with mocked-up book covers and descriptions from each of 42 fiction and non-fiction genres, with one of three possible titles per book randomly assigned. Using author names and photographs, we signaled authors’ race, gender, and age and randomly assigned these combinations to each book presented to our subjects. We then asked subjects to rate their interest in purchasing the book, their evaluation of the author’s credentials, and what amount they were willing to pay for the book.

The experimental design of this study strived to eliminate the potential for proxy-based discrimination by providing book descriptions that detailed the authors’ relevant experience. The large sample and careful distribution of respondents across treatment settings provided sufficient statistical power to detect small differences in consumers’ taste-based preferences. It also allowed for exploration of various types of taste-based discrimination observed in the literature, including discrimination against particular groups, homophily, and pro-social behavior. While the study did detect some significant differences, most were so small as to not be practically meaningful. Thus, our overall finding is of little, if any, discrimination by book consumers based on age, gender, or race. In other words, our study finds no evidence of taste-based preferences by consumers that would rationalize discriminatory treatment against Black or of female authors by publishers on the basis of potential audience reception or profitability.

While our respondents noted the race and gender of the authors presented to them, an author’s gender seemed to have no negative significant bearing on their interest in a book, their evaluation of the author as qualified, or the price they were willing to pay for the book. Respondents also tended to see Millennial authors as having less expertise than older authors. The difference was very small and did not translate into any difference in either interest in purchasing the book or in the price they were willing to pay. Moreover, contrary to the author demographics in publishing, our respondents showed a slight but marked preference for Black authors that translated into a willingness to pay about $0.50 more for their books, or 3.5% more than average. This seemingly small difference in book price may translate into a substantial difference in profitability over a book’s sales lifecycle. This result represents the strongest effect in our experimental findings. Moreover, this preference for Black authors was robust, seemingly unaffected by genre or by the race of the respondent—although Black respondents had a marginally more favorable view of Black authors’ credentials than did others. Thus, in our experiment, consumers showed a willingness to pay more for books by Black authors, a finding that suggests the potential for enhanced profitability across a wide range of genres and not only those related specifically to race and ethnicity, the traditional topics to which Black authors have often been relegated.

Our study has several limitations. First, this is a study of stated preferences and not of actual purchasing behavior. Despite reporting greater interest in books by Black authors and a willingness to pay slightly more for their books, we do not know to what extent these reports would accurately reflect either actual behavior of our respondents or patterns of buying in the book market more broadly. Second, data collection occurred at the height of the Black Lives Matter protests during the summer of 2020. These protests highlighted the grim history and current situation of racial inequality in the U.S. In relation to this experiment, the ongoing national conversation on these issues may have sensitized respondents to issues of race and racism and may have increased conscious or unconscious bias in favor of Black authors and their books.

In addition, while providing greater generalizability than a laboratory experiment, the MTurk sample we used may nonetheless differ from the American population in ways that might bias our results. Berinsky et al. [56] conducted an extensive demographic study on MTurk participants. Their conclusion is that subjects are “younger, … more ideologically liberal … [and] they also appear to pay more attention to tasks than other respondents” where the latter was measured by the need for cognition scale [57] using the ANES 2008 questionnaire [58, page 130]. Thus, our respondent sample, following the pattern of other MTurk samples, may be more liberal than the general population in the U.S. and may thus represent a slightly more liberal portion of the book buying population. Even if this is the case, our results nonetheless suggest a marked distance between the demographic profile of published authors and what a potentially large segment of the market is willing to embrace.

While we find no evidence of discrimination against groups based on age, gender, or race, there is nonetheless the possibility we might find biases against groups at the intersection of these characteristics, for example, against Black women or against older White men. We tested for these interactions (and the data and code are available in our supplemental materials). (We ran random effects models for (a) race × gender, (b) age × gender, (c) race × age, and (d) race × gender × age with and without demographic control variables. No interaction effect was significant at *α* = 5%. Moreover, observed effect sizes even at *α* = 10% were tiny—1/16th of a point on the Likert scale for the responses “Author Credentials” and “Purchase Interest”—and not present at all for the response “Willingness to Pay”.) However, with such small effect sizes, another limitation of this study is its lack of statistical power to explore thoroughly these issues of intersectionality.

Finally, the design of the study attempted to eliminate proxy-based discrimination by presenting books and authors as having been vetted and approved by a traditional publisher. However, in real life, inequality in who gets published and how their books are produced and promoted [6], would limit consumers’ exposure to Black and female authors. Moreover, the existing book market, which also includes books by indie authors, relies on algorithms that may exacerbate existing inequalities in representation, production, and promotion. Thus, in our attempt to eliminate the potential for proxy-based discrimination, we may have unintentionally introduced it, as seeing promotion of a book by an unexpected author may have led respondents to conclude that these authors and their books must be unusually good to make it past the gatekeepers. Whether or not our posing as a publisher enhanced the evaluation of a book and its author, the possibility merely underscores the potential importance of the publishing industry’s signals related to the quality of books and authors to mitigate discrimination against Black and female authors. On the other hand, there is the possibility that our posing as a publisher that only pays $0.10 for market research surveys indicates low budgets and low book quality or even that the presence of a publisher did not signify to our respondents. To the extent that our publishing credential did not enhance evaluation, then our findings also extend to indie publishing. In either case, our findings suggest that should the industry embrace a more diverse pool of authors, consumers would be responsive to their offerings.

While the book industry has predominantly excluded female and Black authors and also tended to relegate them to niche genres, this study suggests that book consumers are interested in and willing to buy books by these authors across many popular genres. Moreover, they do not seem to require any pricing differential in order to do so and, in fact, may be willing to pay slightly more for books by Black authors. We conclude that it is high time the industry pursue greater diversity among authors and that from a market standpoint there is no viable argument against doing so.

## Supporting information

S1 Table All experimental book titles organized by genre. Within genre, the three rows provide the three titles (A, B and C) for the three different cover artworks. The rows are ordered by the cover that appears from left to right in Fig 1 for each of the corresponding genre. Title A corresponds to the title rendered in Fig 1.
(PDF)

S2 Table All experimental book blurbs organized by genre. Within genre, the three covers (A, B and C) indicate the three different cover artworks. The rows are ordered by the cover that appears from left to right in Fig 1 for each of the corresponding genre. Cover B under the Medical genre is the blurb rendered in the example survey elicitation found in Fig 2.
(PDF)

S3 Table Manipulation table for one genre. A detailed manipulation table within one genre-sex-race describing breakdown of generation, cover code, first name, last name and number of subject duplicates per setting.
(PDF)

S1 Data (ZIP)

S1 Text (TXT)
