# Supplementary material for: Do book consumers discriminate against Black, female, or young authors?
Source: PLoS One. 2022 Jun 13;17(6):e0267537. doi: 10.1371/journal.pone.0267537 (PMC9191698; doi:10.1371/journal.pone.0267537)
Supplement: S1 Table — Within genre, the three rows provide the three titles (A, B and C) for the three different cover artworks. The rows are ordered by the cover that appears from left to right in Fig 1 for each of the corresponding genre. Title A corresponds to the title rendered in Fig 1. (PDF) [file pone.0267537.s001.pdf]

Table S1 for article "Do book consumers discriminate against Black, female, or young authors?"

Genre	Title A	Title B	Title C
Business	Blueprint for Profit<br>Setting a Course for Success<br>From Zero to Business Hero	The Road to Success<br>The Winning Formula<br>The Homegrown MBA	The Path to Productivity<br>Sail to Success<br>Armchair MBA
Cooking	How to Cook Anything<br>Cook Your Way to a Better Life<br>Deliciousness in Any Dish	Good Food Guaranteed<br>Perfect Portions<br>Cook Like a Master Chef	Four Simple Rules for Great Food<br>Waste-Free Cooking<br>Cooking Like a Pro
Fantasy	The Dark Saga<br>Sorcerer's Curse<br>Battle for the Dark Forest	The Dark Realm<br>Dragon Flame<br>In the Castle of the Witch	Journey Through Shadows<br>Band of Dragons<br>The Game of Demons
History	The Rise and Fall of Nations<br>White Flight<br>Warriors Throughout History	Moments that Changed History<br>Degentrification<br>Conflicts On and Off the Battlefield	Decisions that Shaped the World<br>The Realtors' Plot<br>Soldiers and Weapons
Medical	Breaking the Code of Silence<br>Big Pharma and the Future of Health<br>An Ounce of Prevention	Secrets My Doctor Should Have Told Me<br>Bitter Pills<br>Surviving the Hospital	What Everyone Should Know about Health Care<br>How Drugs Can Make Us Sicker<br>First, Let Them Do No Harm
Mystery	Buried Secrets<br>Now It's Personal<br>From Brooklyn to Broadway	As the Crow Flies<br>Dead and Buried<br>The Brooklyn Game	A Murder of Crows<br>To Catch a Killer<br>Under the Bridge
Polisci / Law	The Scales of Power<br>Balancing Global Power<br>White Power	Power and Liberty<br>Global Superpowers<br>White Nationalism	Bulletproof Justice<br>Crashing Globalism<br>Backlash Politics
Religion	Talking to Angels<br>Why God Is Always Watching Us<br>Body, Mind, and Spirit	Touching Infinity<br>The End of Days<br>Connecting to Spirit	The Awakening<br>The God Within<br>A Higher Wisdom
Romance	Weekend Boyfriend<br>Last Chance for Love<br>Playboy Holiday	Have Me Committed<br>Messy, Crazy Love<br>Vacation Fling	Small Doses<br>Am I Sorry I Love You?<br>It'll Be a Scorcher
Science	Our Future in Space<br>The Future of Bio-Warfare<br>The Master Code	Traveling to the Stars<br>Lab-Grown Epidemics<br>Written in our Genes	Life on Other Planets<br>Test Tube Terrorism<br>Unlocking Our DNA
Science Fiction	The Andromeda Files<br>The Way Station<br>Alien Overlords	The Void Wraith Chronicles<br>The Last Confederate Soldier<br>The Thrall	The Frontier War<br>The Gatekeepers<br>Wake Up, Humans
Social Science	Disabling Discrimination<br>Learning as Equals<br>Love in a Time of Hate	Rethinking Equality<br>The Equality Effect<br>The Rainbow Revolution	The New Inequality<br>Gender in Schools<br>Coming Out Party
Technology	AI: The Future is Now<br>Guarding Our Data<br>Silicon Valley Upstarts	Thinking Machines<br>Privacy in the Digital Age<br>How Silicon Valley Is Taking Over the World	Are Robots Our Friends?<br>Who Else Knows Your Secrets?<br>Masters of Technology
Thriller	The Mind of a Killer<br>Deadly Games<br>Falling Skies	Interrogation Room<br>In Cold Blood<br>Family Affair	The Copycat<br>In the Line of Fire<br>Blood and Water
