# Supplementary material for: Do book consumers discriminate against Black, female, or young authors?
Source: PLoS One. 2022 Jun 13;17(6):e0267537. doi: 10.1371/journal.pone.0267537 (PMC9191698; doi:10.1371/journal.pone.0267537)
Supplement: S2 Table — Within genre, the three covers (A, B and C) indicate the three different cover artworks. The rows are ordered by the cover that appears from left to right in Fig 1 for each of the corresponding genre. Cover B under the Medical genre is the blurb rendered in the example survey elicitation found in Fig 2. (PDF) [file pone.0267537.s002.pdf]

Table S2 for article “Do book consumers discriminate against Black, female, or young authors?”

Genre	Cover	Blurb
Business	A	Business mogul [Author first name and last name] provides an invaluable blueprint for your path to entrepreneurial success from the very first steps of conceptualizing your venture to winning your first customers, delivering value, and sustaining profit.<br><br>[Author first name]'s clear voice, extensive experience, and easy-to-understand presentation come together to make this book a must-have resource in the library of every budding entrepreneur!
	B	All entrepreneurs and business leaders face similar frustrations — personnel conflict, profit woes, and inadequate growth. After years of helping struggling businesses, award-winning business doctor [Author first name and last name] has developed a foolproof strategy for helping clients navigate past these common maladies to achieve the success they desire.<br><br>Now [Author first name] shares with you the winning formula for achieving the business success you have always imagined.
	C	True leaders aren't made by business schools — they make themselves! Business guru [Author first name and last name] will show you how to go from zero to business hero in this comprehensive guide. Skip the classroom and start learning the real knowledge, skills, and experience you need to succeed.<br><br>[Author first name]'s smart and practical approach will teach you the principles it takes most MBAs a lifetime of trial and error to master.
Cooking	A	Chef [Author first name and last name]'s visionary new master class in cooking distills tens of thousands of hours of professional experience into just four simple rules.<br><br>[Author first name] offers a revolutionary approach that will make even a novice cook a master. By explaining the hows and whys of good cooking, this book will teach and inspire cooks of all skill levels how to be more confident in the kitchen and improvise delicious dishes with any ingredients, anywhere, at any time.
	B	If you're a cook, and tired of that all-too-familiar thud of a half-finished pot of mac and cheese hitting the bottom of your trash can, Chef [Author first name and last name] is ready to expand your recipe repertoire with 100 contemporary, tasty, and perfectly portioned recipes for any occasion that will leave your appetite satisfied and your kitchen waste-free.<br><br>Less wasted food equals less wasted money, and [Author first name]'s appropriate portions and delicious, diverse meals will make you feel like a professional chef! Cook your way to a happier, healthier, tastier life.
	C	Great cooking goes beyond following a recipe — it's knowing how to season ingredients to coax the greatest possible flavor from them.<br><br>Master Chef [Author first name and last name] draws on dozens of leading chefs' combined experience in top restaurants across the country to present the definitive guide to creating “deliciousness” in any dish.

Fantasy	A	The tribe elders say I saved our people from a Shadow raiding party with my special powers. *Special powers — ha!* I know for sure that dumb luck saved us. No matter how much I study the Book of Magic, I can't sustain even the simplest spell to push back the Shadows. I can barely light up a room. But that doesn't stop the elders from naming me the Chosen One. Now they insist I'm our only hope and that it's up to me to light the way and lead our tribe safely from the Shadow Lands. I'm pretty sure we're all going to die.<br><br>Light battles shadow in this rich, epic fantasy from international bestselling author [Author first name and last name]
	B	What is a dragon without flame?<br><br>For generations, the proud dragon warriors of the Last Lake wielded their flames as the last line of defense against the evil Sorcerer of the Outer World — until an evil curse wiped out their forces. Sworn to protect the lake and its people, the few, lone survivors make a stand, only to find they've been robbed of flame. Without their fires, the band of dragons aren't strong enough to fight the evil encroaching on the land. Now they must quest to rekindle their fires or die trying.<br><br>International bestsellng author [Author first name and last name] brings us this exciting tale, sure to become a classic.
	C	A fearless warrior, the Knight of the Black Forest's sole purpose is to eliminate the monsters that plague his realm. Confronted by an unthinkable betrayal, Arik quickly discovers that not all of the creatures he hunts are evil, and not everyone he once trusted is good. How will he know which monsters to battle when the cleverest can disguise themselves with the faces of people he used to love? In a fight that cannot be won by strength and cunning alone, he must look within to conquer his toughest battle yet.<br><br>International bestselling author [Author first name and last name] brings us an epic tale of bravery, beauty, and deceit.

History	A	Award-winning historian [Author first name and last name] takes us on an imaginative, guided tour of the past to show us that the future is in our hands. Far more than a cautionary retelling of events, [Author last name]'s revelatory new history of the world brings to life pivotal choices and moments that have defined cities, nations, and empires.<br><br>In this sweeping and engaging history of both famous and obscure leaders, [Author last name] focuses on key decisions, showing us that the rise and fall of nations is as much about political, economic and social forces as it is about individual choice.
	B	Award winning historian [Author first name and last name] tells the tale of degentrification in the Boston neighborhoods of Dorchester and Mattapan in the mid- to late twentieth century.<br><br>Through firsthand interviews and investigation of private documents and public records, [Author last name]'s revelatory account brings to vivid life a plot by realtors to instigate white flight to the suburbs and to change the racial character of these urban neighborhoods.
	C	From fighting face-to-face to impersonal attacks carried out by drones and computers, war and violent conflict have always been integral to human history. Preeminant military historian [Author first name and last name] takes us on a journey through seminal moments in the history of combat.<br><br>[Author last name]'s authoritative history examines differences in combat techniques and weapons as well as the enduring nature of our causes for war. This unique volume illuminates what it means to be a warrior, both on and off the battlefield.

Medical	A	Written by award-winning investigative journalist [Author first name and last name], this book offers an in-depth investigation about how our health care system really works (and doesn't). Drawing upon hundreds of interviews with doctors and other healthcare providers, [Author last name] offers a friendly, reliable, and timely tutorial that provides answers to the questions we should all be asking but that our healthcare system doesn't want us to ask.<br><br>Covering a range of topics from privacy of healthcare information to medical marijuana, [Author last name] breaks the code of silence and provides us with the information we all need to know to get the best results from our healthcare system.
	B	What happens when medication is a tool for profit rather than for better health? Health science reporter [Author first name and last name] blows the whistle on pharmaceutical industry practices that put profits before patients.<br>Covering everything from clinical trials to production and distribution, [Author last name] reveals that while medical science has opened up a new world of treatments and better health outcomes, the unbridled greed of Big Pharma could actually be making us sicker.
	C	Why are so many people happy with their healthcare solutions but unhappy with their care? In this must-read book, board certified surgeon [Author first name and last name] explains how remarkable innovations in healthcare have led to better and more effective treatment options than ever before, but have only added to the pressures healthcare providers face. In tandem with profit-driven healthcare, these new treatments have reduced the time that doctors spend talking with patients and their capacity to listen to our concerns.<br><br>Dr. [Author last name] provides detailed advice about how you can be a better advocate for yourself and your loved ones to ensure your needs are met in today's fast-paced medical system.

Mystery	A	Rumors have swirled for years about the odd, quiet man who lives at the top of the hill. So when a high school student goes missing, the residents of this small, Southern town immediately finger him as a suspect. Detective Caleb Lacroix knows better than to base a case on rumors, and his investigation soon unearths a truth more shocking than anyone expected.<br><br>International bestselling author [Author first name and last name] will keep you guessing with this clever mystery.
	B	When a neighbor's loving wife is found murdered, it shakes newly promoted police chief John Lee, himself a widower, to the core. He pledges to bring justice to his small town, but when another woman connected to him turns up dead, he realizes the crimes are personal and that catching a killer will require him to dig into a past he'd rather leave dead and buried.<br><br>The past is never far behind us in this tense mystery from international bestselling author [Author first name and last name].
	C	Lizzy's twin sister Sara is a Broadway star who just landed the role of a lifetime, along with a trouble-, some secret admirer. When Sara refuses to meet her new number one fan, he takes his revenge. Lizzy won't rest until Sara's killer is caught, but the only way to lure him out is to pretend he murdered the wrong sister. Can shy Lizzy fool everyone, including Sara's co-stars, long enough to catch a killer?<br><br>*The Phantom of the Opera* meets *Mean Girls* in this Broadway mystery from international bestselling author [Author first name and last name].

<table>
<tr><td>Polisci / Law</td><td>A</td><td>Democracy. Checks and balances. The land of the free. These are the promises of America, but how free and fair is our government, really?

In this shocking book, veteran investigative journalist [Author first name and last name] reveals the subtle and surprising ways the balance of power is being exploited in government and just how closely we tread to becoming a nation without justice.</td></tr>
<tr><td></td><td>B</td><td>Whether or not you know it, globalization has already changed your life. Technological and economic processes have created a world where a decision in Beijing impacts New York before the sun rises, and yet most of us don't understand the world's new interconnectedness or who controls it. As the world comes closer together, are we also being torn apart?

Political scientist [Author first name and last name] investigates the modern superpowers driving globalism and the implications the race for cheaper labor and consumer products is having on the fabric of our local communities.</td></tr>
<tr><td></td><td>C</td><td>Award-winning political scientist [Author first name and last name] investigates the modern rise of the White Power Movement. While the organizations and players involved used to work quietly behind the scenes to influence politics and society, they've taken on a vocal and visible public role in recent years. [Author last name] shows the series of events that have given this hate-based movement a pathway to avoid censure and a platform that speaks to the heart of mainstream White America.

[Author last name] traces this remarkable evolution, showing how identity politics pushed by the Left to promote inclusion and awareness have paved the way for this right-leaning movement that advocates an America for White Americans.</td></tr>
<tr><td>Religion</td><td>A</td><td>What happens when we die? Do sinners go to Hell? Is there a heaven? Revived after dying in a terrible car accident, Reverend [Author first name and last name] awakened with answers to these questions and more about life after death.

In this uplifting book, [Author last name] shares the conversations with the other side that will challenge everything you think you know about religion, death, and the meaning of life. These beautiful messages of Divine love and acceptance will change your life.</td></tr>
<tr><td></td><td>B</td><td>Do you consider yourself a moral person? Would you change your behavior if you truly believed God is always watching and sitting in judgment, deciding not only your future but that of humanity? Renowned theologian [Author first name and last name] explores inner spirituality alongside beliefs about what happens when we die and prophecies for the End of Days.

[Author last name]'s inspiring collection of essays invites us to embrace a personal connection with God that celebrates our inner moral wisdom and our capacity to create a more heavenly world.</td></tr>
<tr><td></td><td>C</td><td>Ancient wisdom provided a blueprint for mind-body health and well-being. The power of that knowledge has not diminished over the centuries, even with advances in modern science and medicine. Medical doctor and Ayurvedic master [Author first name and last name] brings you the essential practices proven to strengthen your mind and body and the connection between them.
Through quizzes and workbook questions, [Author last name] will guide you to develop your own personalized plan for unlocking your higher wisdom, connecting to spirit, and living your healthiest and happiest life.</td></tr>
</table>

Romance	A	A brilliant heart surgeon, Lily knows exactly how to mend a broken heart — as long as it's not her own. She tells herself she's ready for commitment until her ex-flame returns to town. Suddenly unable to to escape the reminders of what happened when she opened herself to loving the wrong man, Lily can't bring herself to take a chance on the one who might just be right — until Carson makes her a crazy proposal. To get out of the friend zone and into her heart, Carson offers to be her weekend boyfriend, keeping things strictly platonic during the week and turning up the heat for a series of weekend affairs. Is his plan to woo her in small doses just the medicine she needs?<br><br>This rompy medical romance from international bestselling author [Author first name and last name] will make you laugh so hard you cry.
	B	If you knew love would hurt, would you fall anyway? If you knew love would die, would you still try? If you knew love would leave you, would you let it go? Cursed with a magical gift to see the future, Ray knows exactly what will happen if he falls for Lucy. But some tragedies are hard to resist.<br><br>From international best-selling author [Author first name and last name] comes a love story that will break your heart and stay with you long after the final chapter.
	C	When Kelly is approached on vacation by the man she believes is a shallow playboy, she figures there's no harm in letting herself have a hot, hot fling. But Rodrigo isn't who he pretends to be. When his secrets put them both in danger, can he convince Kelly that, despite the lies he's been telling, his feelings for her are scorchingly real and that he wants to make their temporary fling permanent?<br><br>International bestselling author [Author first name and last name] delivers a novel so hot the pages will burn your fingers!
Science	A	How will humanity survive climate change? Should we colonize space? Are we alone in the universe? Leading NASA scientist [Author first name and last name] presents the latest scientific discoveries about space, space travel, and life among the stars.<br><br>Accessible and humorous, [Author last name]'s scientific tour de force answers our most pressing questions about the future of humanity and our place in the cosmos.
	B	Throughout history, combat has been carried out in more and more spectacular ways, from the hand-to-hand weaponry of ancient times to the advancements in firearms in the 18th and 19th centuries, and then to the nuclear capabilities of the 20th century. Now as we advance through the 21st century, weaponry has taken a surprising and dramatic turn, and war may soon move from the battlefield to the body.<br><br>Microbiologist [Author first name and last name] takes us inside the secret laboratories where an army of microbes are being developed in preparation for the next major war. Where does the biggest threat of bio-warfare lie and how can we possibly defend ourselves against a threat we can't see?
	C	Long the province of science fiction, designer DNA is quickly becoming a reality. Would you take your own DNA in hand to remake a better you? Geneticist [Author first name and last name] describes the state of modern genetic science and just how close we are to unlocking our master code.<br><br>In the next decade, gene therapies will become increasingly available, and people will have the option to rewrite the destiny written in our genes. But should we?

Science Fiction	A	Mankind's outer colonies are disappearing. Without warning. Without a trace. Fleet command chalks the attacks up to pirates, but Captain Johnson of the Andromeda isn't buying it. Defying command, he leads his misfit crew into hostile territory in search of answers. But none of them are prepared for their encounter with the Void Wraith, an unstoppable foe that was supposed to be the stuff of legend and is now headed for Earth.<br><br>This sci-fi thriller from International bestselling author [Author first name and last name] will keep you at the edge of your seat!
	B	Eustice Williams is not like other humans. Living a secluded life in the backwoods of Montana, he carries a nineteenth-century rifle and never seems to age — a fact that has recently caught the attention of prying government eyes. The truth is, Eustice is the last surviving confederate soldier of the American Civil War and, for over a century, he has operated a secret way station for aliens passing through on journeys to other stars. With the FBI watching him, the gifts of knowledge and immortality that his intergalactic bosses have bestowed upon him are proving to be a nightmarish liability. If his secrets are uncovered, it will mean mankind's impending destruction. Can he keep his secrets safe and save the world from alien invasion?<br><br>International bestselling author [Author first name and last name] delivers a chilling sci-fi thriller about what could happen when mankind learns it's not alone.
	C	They came to Earth and conquered us without firing a shot. We were enslaved without even knowing it. One look into their strange alien eyes, and we experience our deepest desires — along with a blissed-out stupor that makes us completely biddable, even when we're marching to the alien version of a slaughterhouse. There are a few, like me, who can resist the thrall. Can we wake our fellow humans up before our alien overlords make us their next meal?<br><br>International bestselling author [Author first name and last name] will lure you into a richly imagined world where the human drama is simply... delicious.
Social Science	A	An Olympic hopeful now wheelchair bound after an accident. A leading surgeon now debilitated by MS. A manager fired for taking breaks to manage her diabetes. Using personal accounts, sociologist [Author first name and last name] reveals the shortcomings of our public policies and protections, many of which have been aimed at curbing gender and racial discrimination rather than discrimination based on disability.<br><br>[Author last name] weaves together these stories and dozens of others of people who have faced discrimination based on disability, the new frontier of inequality, and suggests a radical new model for approaching discrimination and inequality.
	B	What would happen if boys and girls were given equal opportunity in school? Based on years of research on gender and childhood education, sociologist [Author first name and last name] describes the very real differences in the ways boys and girls learn. As our education system has adopted a one-size-fits-all approach to education under the banner of equal treatment, neither gender is receiving the support needed to succeed.<br><br>[Author last name] reports on the stunning outcomes achieved through curriculum that is not gender-blind but gender-sensitive, meeting the uniquely different needs of boys and girls to give them an equal opportunity to meet their fullest potential.
	C	Anyone observing New York's Gay Pride Parade in 2019 might have been secure in imagining that the rainbow revolution had already occurred, the battle for acceptance fought and won. Indeed there was much to celebrate, including the strides made in winning rights and recognition for the LGBTQ+ community. Yet cultural anthropologist [Author first name and last name] takes us inside this community to show the daily struggles of people denied the equal treatment promised by law but withheld by their neighbors.<br><br>[Author last name]'s touching and insightful ethnography about various kinds of love in a time of bigotry and hatred shows that the revolution isn't nearly over. It is only just beginning.

Technology	A	Are robots our friends? We've eagerly invited Google, Siri, and Alexa into our lives and our homes, embracing the convenience of artificial intelligence (AI). Data scientist [Author first name and last name], a lead designer of the algorithms used by AI to crunch data on our own behaviors and those of millions of other users, vividly describes the process of designing AI sysems enabed to satisfy our whims, whether vacuuming our floors or answering our voice commands.<br><br>But do robots really make our lives better, or are they mascarading as helpers while they steal our privacy?
	B	Think twice before you like that cat video! Every time you interact onto social media or click on a website link, someone's watching and tracking you. [Author first name and last name], an industry insider who has worked for some of the biggest tech firms, uncovers standard industry practices that sound like the stuff of wild conspiracy theories but are the unvarnished truth. These companies have vast amounts of information about you — data that they collect and wield in the name of better marketing, but that could easily be used for monitoring and surveillance.<br><br>They're watching your every move — where you go, what you buy, what you browse, whom you contact. Do you know how to guard yourself?
	C	Silicon Valley is the tech playground for some of the country's brightest minds. Veteran tech writer [Author first name and last name] takes us on a tour of some of the biggest Silicon Valley successes, including Google and Facebook, letting us look inside the daily operations and the process of idea conception from the cubicles to the board rooms.<br><br>Tracing the success and failure of some of their biggest initiatives, [Author last name] shows that what might seem like play to these masters of technology is big business that has the potential to change the world in ways they never intended.

Thriller	A	Homicide detective Jack Haynes always played by the book until a vicious serial murder compelled him to doctor evidence against the suspect he was certain was the killer. His deceit comes back to haunt him when his lie is exposed and another murder is committed, this one close to home. Is it a copycat case, or did he put the wrong man behind bars? Framed for the crime, Jack is running out of time to find the killer and clear his name.<br><br>International bestselling author [Author first name and last name] delivers a nail-biting thriller that will keep you guessing until the last page.
	B	When Navy SEAL Logan Matthews loses his entire team in an ambush on his last day of deployment, he's devastated. When he later learns the attack was not the act of an enemy but of his own government, he begins to unravel a conspiracy that reaches all the way to the president. What those responsible don't realize is that the very same government that betrayed his team trained him to be a deadly weapon, and now he's out for revenge.<br><br>This fast-paced thriller from international bestselling author [Author first name and last name] is full of twists and turns you won't see coming.
	C	When a soon-to-be presidential nominee disappears under the watch of a veteran agent, the by-the-book pro Laurel Johnson explores every possible avenue to get to the bottom of it. As the mystery unravels, she learns that the plot involves her own family. Protecting them would mean breaking every rule she's sworn to uphold. Will she choose duty or family?<br><br>Bestselling international author [Author first name and last name] weaves a tight spell in this psychological thriller you won't want to put down.
