# Supplementary material for: Do book consumers discriminate against Black, female, or young authors?
Source: PLoS One. 2022 Jun 13;17(6):e0267537. doi: 10.1371/journal.pone.0267537 (PMC9191698; doi:10.1371/journal.pone.0267537)
Supplement: S3 Table — A detailed manipulation table within one genre-sex-race describing breakdown of generation, cover code, first name, last name and number of subject duplicates per setting. (PDF) [file pone.0267537.s003.pdf]

Table S1 for article "Do book consumers discriminate against Black, female, or young authors?"

genre	gender	race	generation	cover code (A,B,C)	first name code (1–9)	last name code (1–14)	# subjects per cell
Business	Female	Black	Boomer	A	2	2	6
Business	Female	Black	Boomer	A	3	1	6
Business	Female	Black	Boomer	A	4	14	6
Business	Female	Black	Boomer	B	1	3	6
Business	Female	Black	Boomer	B	5	4	6
Business	Female	Black	Boomer	B	7	5	6
Business	Female	Black	Boomer	C	6	8	6
Business	Female	Black	Boomer	C	8	7	6
Business	Female	Black	Boomer	C	9	6	6
Business	Female	Black	GenX	A	5	9	6
Business	Female	Black	GenX	A	7	11	6
Business	Female	Black	GenX	A	9	10	6
Business	Female	Black	GenX	B	3	13	6
Business	Female	Black	GenX	B	4	14	6
Business	Female	Black	GenX	B	6	12	6
Business	Female	Black	GenX	C	1	1	6
Business	Female	Black	GenX	C	2	2	6
Business	Female	Black	GenX	C	8	3	6
Business	Female	Black	Millennial	A	1	4	6
Business	Female	Black	Millennial	A	2	5	6
Business	Female	Black	Millennial	A	8	6	6
Business	Female	Black	Millennial	B	3	7	6
Business	Female	Black	Millennial	B	6	8	6
Business	Female	Black	Millennial	B	7	9	6
Business	Female	Black	Millennial	C	4	12	6
Business	Female	Black	Millennial	C	5	10	6
Business	Female	Black	Millennial	C	9	11	6
